# Housing and dietary effects on production performance, quality index, and chemical composition of Japanese quail eggs

**DOI:** 10.1016/j.vas.2024.100340

**Published:** 2024-02-15

**Authors:** Md. Amir Hossain, A.S.M. Mahbub, Shah Ahmed Belal

**Affiliations:** a Department of Poultry Science, Khulna Agricultural University, Bangladesh; b Department of Poultry Science, Sylhet Agricultural University, Bangladesh

**Keywords:** Quail egg, Dietary effect, Housing effect, Chemical composition, Egg index

## Abstract

Quail housing and diet significantly impact egg yield and quality. This study assessed the impact of diets and housing arrangements on Japanese quail's egg production, egg index, and quality. In two trials, birds were reared in cages and on the floor with a commercial layer diet (CLD), and an experimental diet (ED) for 32 weeks. Compared to floor-reared birds, cage birds achieved 50% egg production and sexual maturity first. With dietary effects, the CLD diet showed similar results. Furthermore, their feed efficiency, hen house egg production (HHEP), and hen day egg production (HDEP) were significantly higher in cage birds that consumed CLD. Bird livability was unaffected by the housing system, while birds fed CLD had longer lifespans. The housing system had no discernible effects on egg dry matter (DM), crude protein (CP), crude fat (CF), or ash percentage. Nevertheless, the egg albumen's DM, CP, ash%, yolk's DM, CP, and CF were greater in the birds fed CLD. Barring shell thickness, weight, and Haugh unit, birds raised in cages exhibited higher egg exterior index values. Besides the yolk ratio, yolk index, and albumen weight ratio, birds given CLD exhibited enhanced egg interior quality. Ultimately, the most optimal egg production performance, improved egg quality, and prolonged lifespan of Japanese quail were obtained with cage raising and feeding practices utilizing CLD.

## Highlights

• Quails reared in cages and fed a commercial layer diet showed superior egg production and quality.
• Housing did not affect egg composition, but the commercial layer diet resulted in higher values for several egg components.
• Cage-raised birds exhibited higher exterior egg quality indices, except for shell thickness, weight, and Haugh unit.
• The study concluded that cage raising and feeding with a commercial layer diet resulted in optimal egg production, improved egg quality, and longer lifespan for Japanese quail.

## 1 Introduction

Providing safety for birds, whether in the feed or the environment, is the overall trend of the poultry industry (Alagawany et al., 2019; Elnesr et al., 2019; Ismail et al., 2021; Reda et al., 2020). A successful poultry farming enterprise depends on housing and nutrition. Housing systems have a significant impact on poultry products and their quality. Every housing scheme offers benefits and drawbacks regarding the bird's performance, health, and welfare. The proper housing system and diet for layer chickens should be considered to increase egg production and quality attributes. The different housing arrangements may impact laying hens performance and production indices, including feed consumption, feed efficiency, egg weight, and egg output (Batkowska et al., 2014).

Over the past decade, there has been increasing attention on animal welfare in poultry farming practices worldwide. This has led to modifications of traditional cage systems to non-cage systems or enriched cages (Batkowska et al., 2014). Housing is considered one of the most significant non-genetic elements affecting hens' production capacity, health, behavioral, productive, and reproductive features (Roshdy et al., 2010). Hence, in order to meet their needs and enable them to realize their genetic potential, laying hens should be given enough nutrients by modifying the diet density with feed intake (Leeson & Summers, 2005). However, studies have shown that rearing procedures have little impact on egg production performance of current layers (Ahammed et al., 2014), possibly due to various factors such as age, genotypes, environment, housing systems, and diet (Rakonjac et al., 2014; Steenfeldt & Hammershøj, 2015).

Several housing systems are used in poultry production, notably cage housing systems (CRS) and floor-litter housing systems (LRS) (Wan et al., 2021). While research on the effects of housing systems is rich for chicken, it is scarce for the Japanese quail industry. Quails are typically raised in multi-tiered cages during the growing and laying seasons, but they can also be raised on floors with no impact on egg-laying ability (Padmakumar et al., 2000). Japanese quails raised on deep litter have been found to lay eggs and reach 50 % egg production later than those raised in cages, with considerably higher egg output and hatchability percentages (Kundu et al., 2003). However, according to Elsayed and Gharib (2019) when compared to the deep litter system with coarse sawdust, the egg quality (both internal and exterior features) in cage systems and deep litter systems with fine sawdust was better. Egg quality depends on several characteristics crucial to the world's egg industry and is influenced by various elements, such as the food and age of the hen (Kaczmarek et al., 2016). It is well established that a rise in dietary protein consumption during the production phase is linked to an increase in egg size (Gunawardana et al., 2008). However, some studies reported that housing systems had no impact on specific external and internal egg features, but feed intake and conversion were much higher in cages than on the floor (Rouf et al., 2015).

A hen's diet has impact on the nutritional content of her eggs, but her environment of raising, strain, and age also play a role (Heflin et al., 2018). Eggs are a rich source of important proteins, lipids, vitamins, minerals, and bioactive substances, and the compositions and net amount of these nutrients may vary depending on the food, and environmental factors of the hens (Kuang et al., 2018). There is a widespread misconception that eggs from hens kept in cage systems are less nutritious than eggs from free-range chickens (Bejaei et al., 2011). Free-range eggs may contain more n-3 fatty acids due to the foraging behavior of the chickens (Anderson, 2011), but they may not always offer significant nutritional improvements over eggs produced by battery-caged hens (Hidalgo et al., 2008). Egg chemical composition can be altered by production system changes, with alternative production methods affecting egg quality and chemical composition (Vlčková et al., 2019). For example, eggs from organically reared hens exhibited lower levels of dry matter, proteins, and lipids than those produced using the cage method (Rakonjac et al., 2014).

Studies have provided little and frequently contradictory information about macro and micro nutrients of eggs produced by hens raised in free-range environments. One study found that free-range hens had lower zinc concentrations than commercially raised chickens raised in conventional and organic housing systems, likely due to the ingestion of soil and grasses (Giannenas et al., 2009). Another study found that despite consuming appropriate food, chickens kept in free-range environments produced eggs with lower quantities of phosphorus and zinc, indicating differences in mineral composition between organically and conventionally raised chicken eggs (Küçükyılmaz et al., 2012). Cage rearing is often favored in the poultry industry, especially in developed nations where around 90 % of chickens are raised in cages (Tauson, 1998). This method increases production for economic gain, but unfortunately, animal welfare is often not taken into consideration, despite the fact that laying eggs in nest on floor is a maternal behavior. In contrast, quail hens tend to lay their eggs in nests, and nests account for a significant percentage of eggs compared to artificial shelters and floors covered in plants (Schmid & Wechsler, 1997).

However, less focus has been on the combined impact of housing and diet on production performance, quality, chemical composition of Japanese quail eggs. Therefore, the main goal of this current study is to address this gap in research.

## 2 Materials and methods

### 2.1 Animals and experimental design

**Fig. 1:**
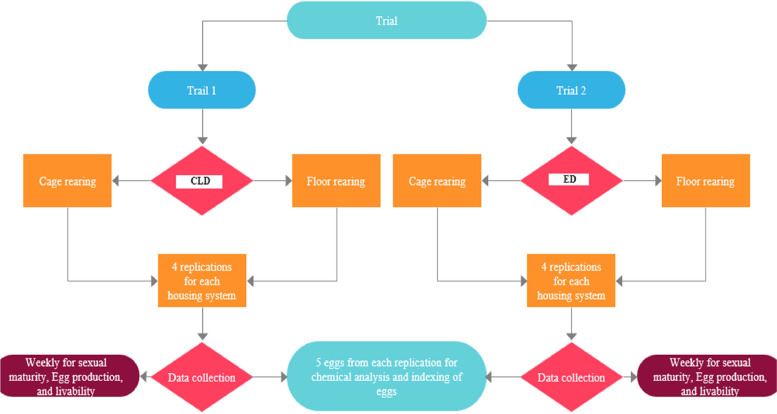
Flow chart of the entire experiment.

Japanese quail hens were used in the experimental design, which was split into two distinct trials known as Trial 1 and Trial 2. These tests were carried out in two different housing systems—the cage and the floor—that were set up concurrently inside the same building. To provide a thorough understanding of the dietary implications inside each housing system, two different diets, namely commercial layer diet CLD and experimental diet (ED), were also supplied under each of these housing settings. A total of 450 day old quail chicks were raised in a brooder at the Youth Training Center in Sylhet, Bangladesh, under uniform lighting conditions of 22 to 24 h and temperature conditions of 37 °C and reduced approximately 2.7 °C until they reached four weeks of age. Subsequently, 400 birds were randomly allocated to the cage and floor systems, with each rearing system consisting of four replications/diet and each replication containing 25 birds (Fig. 1), and the rest of the birds also randomly selected and sent to a commercial farm. The trial employed a suitable sample size because significant results in quail bird research require a sufficient sample size to ensure statistical significance, accuracy, resource efficiency, ethical considerations, and generalizability. The temperature was consistently maintained at an average of 25–28 °C, and the birds were exposed to 14–18 h of light per day from the fifth week until the end of the study.

### 2.2 Housing

The cage, designed explicitly for this study, was fabricated using welded mesh and was composed of four sections, each measuring 120 x 60 x 30 cm. The dimensions of each compartment were tailored to house 40 birds on an eight sq. ft floor, which provided ample space for the birds to move around comfortably. The cage's sides, top, and floors were all constructed from welded mesh, with dimensions of 2.5 x 5.0 cm and 1.25 x 2.5 cm, respectively, ensuring adequate ventilation and optimal air circulation. A gentle gradient was incorporated into the floor to facilitate the egg collection process, extending to an outer extension of welded mesh measuring 7 cm in length, designated as the egg roll. Linear feeders and waterers were installed throughout the compartment's length and width to ensure uniform access to feed and water. Removable fecal pans were strategically placed between the two tiers to enable the accessible collection of fecal matter, thereby maintaining hygiene standards. In the floor-raising system, each bird was allocated an individual space of 151 cm². A 3″ (three-inch-deep) litter was placed on the ground for the birds' comfort. Given that birds raised on sawdust exhibited superior growth performance, welfare, and the lowest frequencies of feather pecking. Furthermore, sufficient waterers and feeders were provided to ensure the birds' well-being.

### 2.3 Diet

Two types of mash feed were employed in our study: ED, which was prepared in accordance with the National Research Council's (NRC, 1994) laying quail management standards to cover all nutrient needs (Tables 1 & 2), and CLD, which was commercial feed mill's ready-made layer quail diet (Alaya group) (Tables 3 & 4).

**Table 1:** Percentage composition of different ingredients of the quail diet.

Ingredients	Percentage
Corn	56.5
Fish Meal	2.00
Soy Bean Meal	27.05
Wheat offal	3.00
Bone Meal	2.50
Vegetable oil	1.50
Di-calcium phosphate	2.00
Na Cl	0.40
Limestone	4.30
Methionine	0.25
Lysine	0.25
Vitamins and Minerals Mixture	0.25
Total 100	100

**Table 2:** Calculated analysis of the quail diet.

Nutrients	Amount
Metabolizable Energy	2910 K.cal
Crud Protein	20.34 %
Calcium	2.0 %
Phosphorus	0.58 %
Lysine	1.15
Methionine	0.5

**Table 3:** Percentage composition of different ingredients of commercial layer diet.

Ingredient	Quantity (kg/100 kg)
Maize	50
Soybean Meal	10.9
Wheat Offal	12
Limestone	8.9
Bone Meal	1.9
Groundnut Cake	15
Layer Premix	0.25
Toxin Binder	0.15
Salt	0.3
Vitamin-Minerals Premix	0.25
Methionine	0.2
Lysine	0.15
**Total**	**100**

**Table 4:** Nutrients of Commercial Layer Diet.

Nutrients	Amount
Metabolizable Energy	2900 K.cal
Crud Protein	20.7 %
Fat	2.5 %
Fiber	4.5 %
Calcium	2.5 %
Phosphorus	0.55 %
Lysine	1.0 %
Methionine	0.4 %

### 2.4 Weight and metric analysis

A total of eighty (80) eggs were randomly selected (five (5) from each replication/ twenty (20) for each dietary trial/forty (40) from each housing system), and subjected to daily measurements. An electrical precision balance with a precision of 0.001 g was utilized to measure the weight of the eggs along with their constituent parts, including albumen, yolk, and eggshells with membranes. The dimensions of egg length and width (mm), eggshell thickness (mm), and albumen and yolk height and width (mm) were measured through the employment of an electronic caliper. Haugh Unit were measured by using Haugh Tester.

Production performance was calculated by using the following formulas,

$$Hen\;day\;egg\;production\;\left(HDEP\right)\;\%\;=\frac{Total\;egg\;number}{Daily\;hen\;number\;}\times 100$$

$$Hen\;house\;egg\;production\;\left(HHEP\right)\;\%\;=\frac{Total\;number\;of\;eggs\;laid\;on\;a\;day}{Total\;number\;of\;hens\;housed\;at\;the\;beginning\;of\;laying\;period}\times 100$$

$$Feed\;efficiency\;\left(\mathrm{per}\;\mathrm{kg}\;\mathrm{egg}\;\mathrm{mass}\right)\;=\frac{Kg\;of\;feed\;intake}{Kg\;of\;egg\;produced\;}$$

$$Livability\;\%\;=\frac{No.\;of\;live\;chicks\;up\;to\;specified\;time}{Number\;of\;placed\;chicks\;}\times 100$$

Egg quality (External and internal qualities) were calculated by using the following formula,

$$Egg\;shape\;index\;=\frac{Egg\;width}{Egg\;length\;}\times 100$$

$$Egg\;surface\;area\;{\left(cm\right)}^{2}:\;calculated\;as\;3.9782W^{0.7056},\;where\;W\;=\;egg\;weight$$

$$Eggshell\;density\;{\left(\frac{mg}{cm}\right)}^{2}=\frac{Shell\;weight\;\left(mg\right)}{Egg\;surface\;area\;}\times 100$$

$$Yolk\;weight\;ratio\;\left(\%\right)\;=\frac{Yolk\;weight\;\left(g\right)\;}{Egg\;weight\;\left(g\right)\;}\times 100$$

$$Albumen\;weight\;ratio\;\left(\%\right)\;=\frac{Albumen\;weight\;\left(g\right)\;}{Egg\;weight\;\left(g\right)\;}\times 100$$

$$Yolk\;index\;\left(\%\right)\;=\frac{Yolk\;height\;\left(mm\right)}{Yolk\;diameter\;\left(mm\right)\;}\times 100$$

$$Haugh\;Unit\;=\;100\;log\;\left(albumen\;height\right)\;\left(mm\right)\;+\;7.57\;-\;1.7\;x\;egg\;weight\;{\left(g\right)}^{0.37}$$

### 2.5 Laboratory analysis

#### 2.5.1 Chemical analysis of Japanese quail egg

At the end of the study, eighty (80) samples of albumen, and yolk were randomly collected for chemical analysis in the accredited research lab of the Poultry Science Department at Sylhet Agricultural University. The analysis of DM%, crude protein (CP)%, crude fat (CF)%, and ash% contents was performed by the classical method of chemical analysis by using an Oven, Kjeldahl apparatus, Soxhlet extractor, and Muffle furnace, respectively.

### 2.6 Data collection and analysis

Data obtained from the experiment, such as age at sexual maturity and 50 % egg production, hen day egg production, hen housed egg production, feed intake, feed efficiency, mortality, and egg chemical analysis, were entered into MS Excel spreadsheet. The dataset was checked for missingness and integrity by careful visual examination of the spreadsheet. The continuous variables were checked for normal distribution using the visual observation of the histogram. The effects of the treatments on the measured traits were analyzed using a univariate generalized linear model. The model included the outcome of interest as the dependable variable, treatments as the fixed effect, and replication as the random effect. The mean was reported as the least squares mean and their standard error. Differences between treatment means were tested for statistical significance and were adjusted according to the multiple comparison test using Bonferroni corrections. The statistical analysis software used SPSS v 26 for Windows (SPSS, Inc., Chicago, IL). The 'ggplot2′ package from the open-source program RStudio (v1.1.453, RStudio, Inc.) was used to create graphs.

## 3 Results

### 3.1 Age at sexual maturity and 50 % egg production

Results showed that quails raised in cage reached sexual maturity earlier (*p <* 0.05) and attained 50 % egg production sooner (*p <* 0.05) than those grown in floor rearing environments (Table 5). Furthermore, dietary analysis indicated that quails raised in cage and floor and fed with CLD had faster sexual maturation and earlier attainment of 50 % egg production (*p <* 0.042) than those fed with the ED (Table 5).

**Table 5:** Housing and dietary effects on age at sexual maturity and 50 % egg production.

	Age at sexual maturity (days)	Age at 50 % egg production (days)
Housing effects with the experimental diet		
Cage rearing	38.85	78.60
Floor rearing	40.72	80.42
SEM	0.46	0.431
Significance	⁎⁎	⁎⁎
Housing effects with commercial layer diet		
Cage rearing	37.97	76.92
Floor rearing	38.85	78.35
SEM	0.207	0.356
Significance	⁎⁎	⁎⁎
Diet effects on cage		
Experimental diet	38.85	78.6
Commercial layer diet	37.97	76.92
SEM	0.195	0.374
Significance	⁎⁎	⁎⁎
Diet effects on floor		
Experimental diet	40.72	80.42
Commercial layer diet	38.85	78.35
SEM	0.47	0.422
Significance	⁎⁎	⁎⁎

⁎⁎ *p <* 0.05, ED=Experimental diet, CLD= Commercial Layer Diet, Cage rearing, Floor rearin*g*.

### 3.2 Laying performance

#### 3.2.1 Hen day egg production performance

**Fig. 2:**
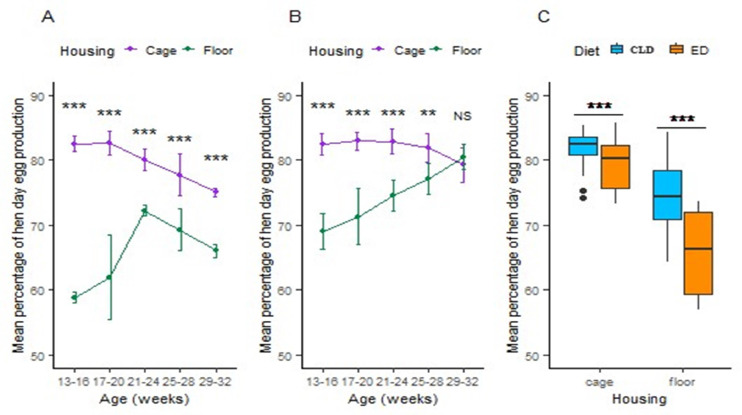
(**A**) Housing effects on hen day egg production performance with the ED, (**B**) Housing effects on hen day egg production performance with CLD, and (**C**) Dietary effect on mean hen day egg production performance in two different housing systems. The values are means ± standard error of the mean. Significance level: ***P < 0.01 = highly significant, **p < 0.05 = significant, NS = non-significant. Abbreviations: ED, Experimental diet; CLD, commercial layer diet.

In trial 1 initial HDEP performance were greater in cage (82.82 %) than those of the floor housing system (58.64 %). Nevertheless, the HDEP percentages for the floor system had dropped to 66.04 % and the cage system to 75.01 % by the end of the study (Fig. 2(A)). In the trial 2, the cage-rearing approach outperformed the floor-rearing system in terms of HDEP performance for the 13–24 weeks period. But starting at 25 to 28 weeks, the floor-reared quail's HDEP performance grew significantly, and by the end of the research, it had surpassed the cage-reared quail's. At 28–32 weeks of age, the two system's HDEP performance differences were negligible (Fig. 2(A)). The both rearing systems demonstrated a highly significant difference (*p <* 0.001) in the mean HDEP performance of Japanese quail fed with CLD compared to the other system. The ED group's HDEP performance in cage was 79.55 %, while the CLD group's was 81.84 %. Similiarly the ED group's HDEP performance in the floor-rearing system was 65.67 %, whereas the CLD group's was 74.48 % (Fig. 2(A)).

#### 3.2.2 Hen housed egg production

**Fig. 3:**
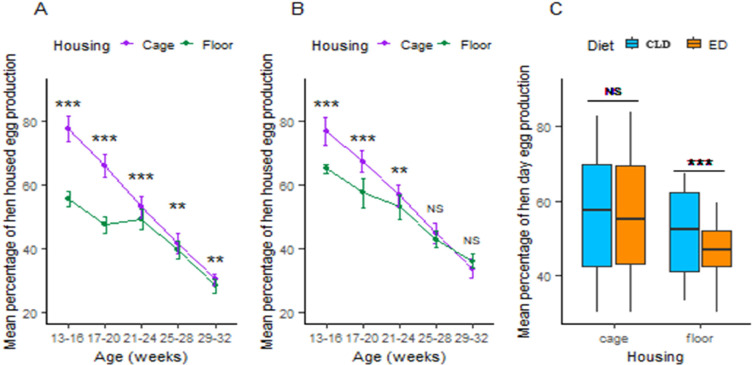
(A) Housing effects on hen house egg production performance in the ED trial, (B) Housing effects on hen house egg production performance in the CLD trial, and (C) Dietary effect on mean hen house egg production performance in two different housing systems. The values are means ± standard error of the mean. Significance level: ***P < 0.01 = highly significant, **p < 0.05 = significant, NS = non-significant. Abbreviations: ED, Experimental diet; CLD, commercial layer diet.

In the first trial, HHEP percentages declined over time in both rearing systems, but performance being higher in cages than on the floor at all phases. By the end of the trial, there was a significant reduction in the disparity between the two rearing systems with percentages of 30.32 % in cages and 28.35 % on the floor (Fig. 3(A)). The second trial showed a significant beginning difference in HHEP performance between cages and the floor. But, by the study's conclusion, this difference had lost its significance (Fig. 3(B)). Furthermore, the study examined the differences in HHEP performance between the ED and CLD trials in both rearing systems. In cages, the difference in HHEP performance between the two diets was not statistically significant; however, on the floor, the CLD trial demonstrated a higher HHEP performance (Fig. 3(C)).

### 3.3 Feed consumption

**Fig. 4:**
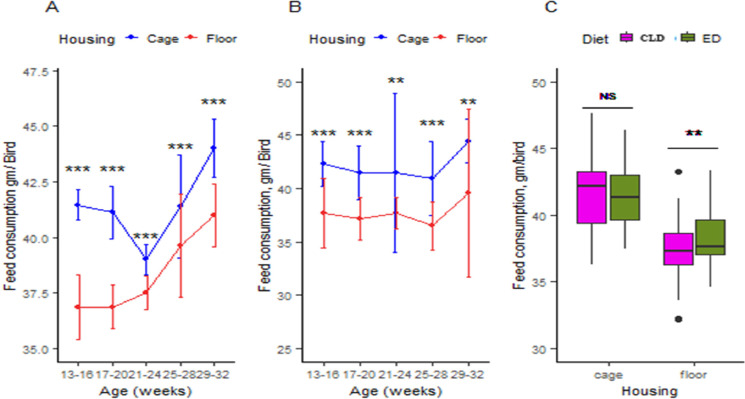
(A) Housing effects on feed consumption in the ED trial, (B) Housing effects on feed consumption in the CLD trial, and (C) Dietary effects on mean feed consumption in two different housing systems. The values are means ± standard error of the mean. Significance level: ***P < 0.01 = highly significant, **p < 0.05 = significant, NS = non-significant. Abbreviations: ED, Experimental diet; CLD, commercial layer diet.

In the ED trial, the feed consumption of Japanese quail housed in cages was 41.46, 41.11, 39.01, 41.39, and 44.00 g/bird for 13–16, 17–20, 21–24, 25–28, and 29–32 weeks of age, respectively, while in the floor rearing system, it was slightly lower at 36.85, 36.87, 37.52, 39.61, and 40.96 g/bird (Fig. 4. (A)). In Trial 2, the feed consumption in cage was 42.30, 41.48, 41.46, 40.93, and 44.40 g/bird, and on floor, it was 37.69, 37.18, 37.67, 36.48, and 39.54 g/bird with CLD, respectively, for 13–16, 17–20, 21–24, 25–28, and 29–32 weeks of age (Fig. 4. (B)). The difference in feed consumption between cage and floor was highly significant (*p <* 0.01) for both diets, except at 21–24 and 29–32 weeks of age in the CLD trial, where the difference was significant (*p <* 0.05). Furthermore, the mean difference in feed consumption between ED and CLD trials in cages remained insignificant, but in floor-rearing systems, the difference was significant (*p <* 0.05) with values of 38.36 and 37.71 g/bird for ED and CLD, respectively (Fig. 4. (C)).

### 3.4 Feed efficiency (Per kg egg mass)

**Fig. 5:**
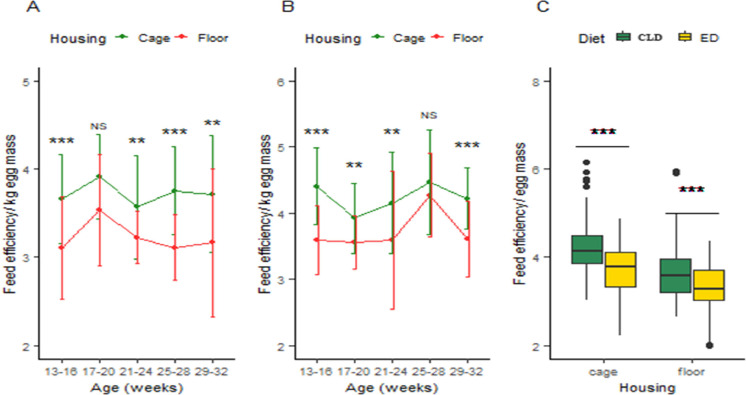
(A) Housing effects on feed efficiency/kg egg mass in the ED trial, (B) Housing effects on feed efficiency/kg egg mass in the CLD trial, and (C) Dietary effects on mean feed efficiency/kg egg mass in two different housing systems. The values are means ± standard error of the mean. Significance level: ***P < 0.01 = highly significant, **p < 0.05 = significant, NS = non-significant. Abbreviations: ED, Experimental diet; CLD, commercial layer diet.

The results of the experiment revealed that the feed efficiency/kg egg mass in the ED trial on cage ranged from 3.56 to 3.91, while on the floor, it ranged from 3.11 to 3.53 at different ages (13–16, 17–20, 21–24, 25–28, and 29–32 weeks). The difference between cage and floor housing systems was found to be highly significant (*p <* 0.001) at the beginning and end of the period but remained significant (*p <* 0.05) at 17–20 and 25–28 weeks of age (Fig. 5(A)). Furthermore, in the CLD trial, the feed efficiency/kg egg mass in cage ranged from 3.92 to 4.46, while on the floor, it ranged from 3.55 to 4.26 at different ages. The difference between the two housing systems was significantly higher (*p <* 0.01) initially and at the end of the experiment and remained significant (*p <* 0.05) from 17 to 24 weeks of age (Fig. 5(B)). Moreover, the effects of ED and CLD on feed efficiency in cage were found to be 3.72 and 4.23, respectively, while in floor housing systems, the feed efficiency was 3.22 and 3.72 in ED and CLD, respectively. Additionally, in both housing systems, the difference in feed efficiency between ED and CLD was highly significant (*p <* 0.01) (Fig. 5(C)).

### 3.5 Livability

**Fig. 6:**
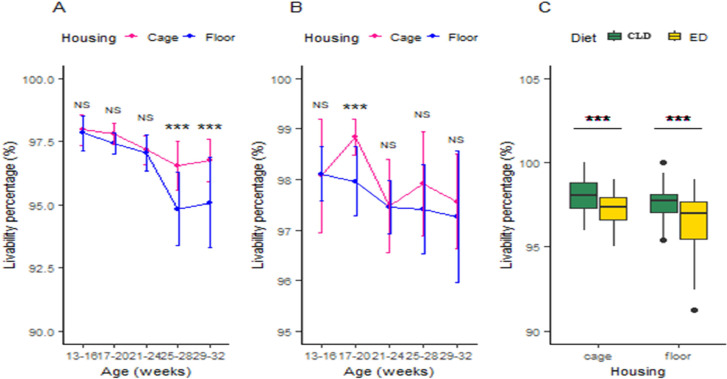
(A) Housing effects on livability percentage in the ED trial, (B) Housing effects on livability percentage in the CLD trial, and (C) Dietary effects on livability percentage in two different housing systems. The values are means ± standard error of the mean. Significance level: ***P < 0.01 = highly significant, **p < 0.05 = significant, NS = non-significant. Abbreviations: ED, Experimental diet; CLD, commercial layer diet.

The experiment results indicate that in the ED-based trial, there was no significant difference in livability percentage between the two housing systems from 13 to 24 weeks. However, from 25 weeks until the end of the study, the livability percentage was significantly higher in the cage system compared to the floor system, with values of 96.53 % and 96.75 % for cage and 94.83 % and 95.08 % for floor at 25–28 and 29–32 weeks, respectively Fig. 6. (A). In the CLD trial, there was no significant difference in livability percentage throughout the entire period, except at 17–20 weeks of age, where the difference between cage and floor remained highly significant (*p <* 0.01) (Fig. 6. (B)). Moreover, the survivability percentage for ED and CLD was 97.24 % and 97.97 % in cage, respectively, and 96.44 % and 97.64 % on floor, respectively (Fig. 6. (C)).

### 3.6 Egg chemical analysis

**Fig. 7:**
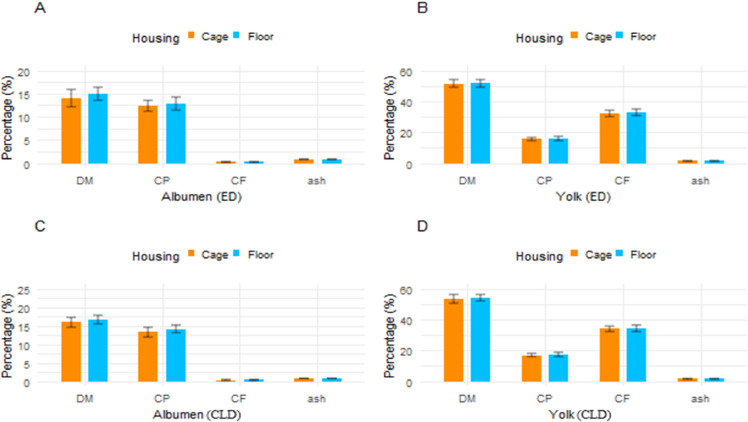
(A) Housing effects on the chemical composition of albumen in the ED trial, (B) Housing effects on the chemical composition of yolk in the ED trial, and (C) Housing effects on the chemical composition of albumen in the CLD trial, (D) Housing effects on the chemical composition of yolk in the CLD trial. The values are means ± standard error of the mean. Abbreviations: ED, Experimental diet; CLD, commercial layer diet.

**Fig. 8:**
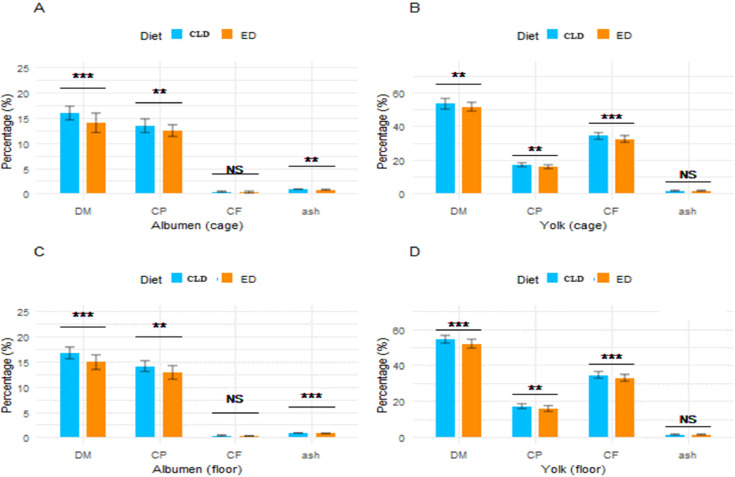
(A) Dietary effects on the chemical composition of albumen in cage, (B) Dietary effects on the chemical composition of yolk in cage, (C) Dietary effects on the chemical composition of albumen in floor, (D) Dietary effects on the chemical composition of yolk in floor. The values are means ± standard error of the mean. Significance level: ***P < 0.01 = highly significant, **p < 0.05 = significant, NS = non-significant. Abbreviations: ED, Experimental diet; CLD, commercial layer diet.

In ED trial the percentages of DM, CP, CF, and Ash in egg albumen were 14.05, 12.44, 0.27, and 0.75 %, respectively, in cage, and 14.90, 12.88, 0.26, and 0.77 %, respectively, in floor (Fig. 7. (A)). The DM, CP, CF, and Ash% in egg yolk were also determined and found to be 51.45, 15.86, 32.34, and 1.54 %, respectively, in cage, and 51.75, 75.96, 32.88, and 1.49 %, respectively, in floor-raised quail eggs (Fig. 7(B)). Similarly, in the CLD trial, the composition of egg albumen, including DM, CP, CF, and Ash%, was analyzed. The percentages of DM, CP, CF, and Ash were found to be 16.00, 13.35, 0.27, and 0.80 %, respectively, in cage(Fig. 7. (C)), and 16.73, 14.02, 0.33, and 0.82 %, respectively, in floor-raised quail eggs (Fig. 7. (D)). Results also showed that in cage, albumen DM, CP, and Ash% were significantly higher (*p <* 0.01) in CLD compared to ED, except for CF% (Fig. 8. (A)). Conversely, in floor, all chemical composition parameters were significantly higher (*p <* 0.01) in CLD compared to ED (Fig. 8. (C)). Regarding yolk, DM% in cage was higher (*p <* 0.05) in CLD trial than in ED, while CP% and CF% showed a highly significant difference (*p <* 0.01) between the two dietary groups (Fig. 8. (B)). Similarly, in floor, except for Ash%, the rest of the three parameters, namely DM, CP, and CF%, were significantly higher (*p <* 0.01) in the egg yolk obtained from CLD trial (Fig. 8. (D)).

### 3.7 Egg index

#### 3.7.1 External qualities of egg

The weight of the eggs produced by cage housing (15.29 g) was greater than that of floor rearing (12.00 g) when fed ED. Nonetheless, the thickness of the shell was somewhat greater during floor raising (0.22 mm) compared to cage rearing (0.21 mm). In addition, the percentage of egg shells in cages was higher (10.15 %) than in floor raising (8.80 %). Cage raising produced larger egg weights (16.22 g) than floor rearing (12.18 g) when the CLD was applied. Compared to cage raising, the thickness of the shell was somewhat greater (0.23 mm) in floor rearing. (0.19 mm). When raising birds in cages (9.18 %) or on the floor (9.20 %), the egg shell ratio percentages were nearly identical. The housing arrangement and food have a major impact on the exterior quality of Japanese quail eggs, according to the study. When compared to floor housing, eggs raised in cages using both diets (ED) typically had better qualities. Floor housing, however, displayed marginally superior shell thickness. For both housing systems, the CLD did better than the ED in the majority of parameters (Table 6).

**Table 6:** Housing and dietary effects on external qualities of Japanese quail egg.

	Egg Weight (g)	Egg length (mm)	Egg width (mm)	Shell thickness (mm)	Shell weight (g)	Egg Shape index	Egg shell ratio%	Egg surface area (cm^2^)	Egg Shell Density (mg/cm^2^)
Housing systems									
Cage rearing × ED	15.29	31.36	24.41	0.21	1.05	78.19	10.15	27.25	46.11
Floor rearing × ED	12.00	31.24	24.37	0.22	1.54	77.78	8.80	22.97	45.95
SEM	0.16	0.08	0.07	0.01	0.02	0.31	0.08	0.28	0.18
Significance	⁎⁎⁎	NS	NS	⁎⁎	⁎⁎⁎	NS	⁎⁎⁎	⁎⁎⁎	NS
Housing systems									
Cage rearing × CLD	16.22	35.19	28.63	0.23	1.54	81.41	9.18	28.40	54.53
Floor rearing × CLD	12.18	30.79	23.85	0.21	1.11	77.50	9.20	28.29	48.18
SEM	0.20	2.39	0.22	0.18	0.03	0.31	0.32	0.14	1.22
Significance	⁎⁎⁎	⁎⁎⁎	⁎⁎⁎	NS	⁎⁎⁎	⁎⁎⁎	NS	NS	⁎⁎⁎
Diet									
ED × Cage rearing	15.29	31.36	24.41	0.21	1.05	78.19	10.15	27.25	46.11
CLD × Cage rearing	16.22	35.19	28.63	0.23	1.54	81.41	9.20	28.40	54.53
SEM	0.11	0.19	0.20	0.02	0.02	0.29	0.23	0.13	0.45
Significance	⁎⁎⁎	⁎⁎⁎	⁎⁎⁎	⁎⁎	⁎⁎	⁎⁎⁎	⁎⁎	⁎⁎	⁎⁎⁎
Diet									
ED × Floor rearing	12.00	31.24	24.37	0.22	1.11	77.50	8.80	22.97	45.95
CLD × Floor rearing	12.18	30.79	23.85	0.36	1.54	77.78	9.20	28.40	48.18
SEM	0.05	0.07	0.07	0.18	0.03	0.37	0.23	0.27	1.18
Significance	NS	⁎⁎⁎	⁎⁎⁎	NS	⁎⁎⁎	NS	NS	⁎⁎⁎	NS

⁎⁎⁎ *p <* 0.01,.

⁎⁎ *p <* 0.05, ED=Experimental diet, CLD= Commercial Layer Diet, Cage rearing, Floor rearing.

#### 3.7.2 Internal qualities of egg

Internal qualities of cage reared Japanese quail eggs under ED showed that the albumen weight (5.79 g), albumen ratio (55.82 %), albumen height (8.07 mm), albumen weight (5.66 g), albumen ratio (37.22 %), and Haugh unit (93.69) were all greater than those of floor rearing. In contrast, floor housing resulted in somewhat larger yolk height (9.08 mm) and yolk diameter (21.61 mm). A comparison between floor rearing and cage rearing using CLD revealed that the former produced eggs with a lower Haugh unit (93.84), albumen weight (5.93 g), albumen height (8.43 mm), albumen diameter (24.41 mm), yolk ratio (34.81 %), and yolk index (42.90 %) (Table 7).

**Table 7:** Table: Housing and dietary effects on internal qualities of Japanese quail egg.

	Yolk weight (g)	Yolk height (mm)	Yolk diameter (mm)	Yolk ratio	Yolk index**%**	Albumen Weight (g)	Albumen height (mm)	Albumen Weight Ratio	Haugh unit
Housing systems									
Cage rearing × ED	5.66	13.44	24.51	37.22	54.86	8.07	5.79	55.82	93.69
Floor rearing × ED	4.23	9.08	21.61	35.38	42.13	6.71	3.91	52.62	85.68
SEM	0.07	0.24	0.15	0.30	0.82	0.10	0.11	0.35	0.53
Significance	⁎⁎⁎	⁎⁎⁎	⁎⁎⁎	⁎⁎⁎	⁎⁎⁎	⁎⁎⁎	⁎⁎⁎	⁎⁎⁎	NS
Housing systems									
Cage rearing × CLD	5.61	8.43	24.71	34.81	42.90	13.76	5.93	55.60	93.84
Floor rearing × CLD	4.25	6.89	21.33	34.05	44.74	9.14	4.13	55.75	85.29
SEM	0.06	0.10	0.16	0.29	0.53	0.25	0.12	0.42	0.57
Significance	⁎⁎⁎	⁎⁎⁎	⁎⁎⁎	NS	NS	⁎⁎⁎	⁎⁎⁎	NS	⁎⁎⁎
Diet									
ED × Cage rearing	5.61	8.43	24.51	34.22	42.86	8.07	5.79	55.82	93.69
CLD × Cage rearing	5.66	13.44	24.71	37.81	54.90	13.76	5.93	55.60	93.84
SEM	0.04	0.26	0.04	0.38	0.84	0.28	0.08	0.32	0.45
Significance	NS	⁎⁎⁎	⁎⁎	⁎⁎⁎	⁎⁎⁎	⁎⁎⁎	NS	NS	NS
Diet									
ED × Floor rearing	4.23	6.89	21.33	35.04	42.13	6.71	3.87	52.62	85.29
CLD × Floor rearing	4.25	9.08	21.61	35.37	44.74	9.14	4.13	55.75	85.68
SEM	0.01	0.13	0.09	0.18	0.48	0.14	0.11	0.44	0.35
Significance	NS	⁎⁎⁎	NS	NS	⁎⁎⁎	⁎⁎⁎	NS	⁎⁎⁎	NS

⁎⁎⁎ *p <* 0.01,.

⁎⁎ *p <* 0.05, ED=Experimental diet, CLD= Commercial Layer Diet, Cage rearing, Floor rearing.

## 4 Discussion

In current study housing conditions have been shown to influence the age at sexual maturity and 50 % egg production of Japanese quail. The results indicated cage-reared quails attained early sexual maturity and reached 50 % egg production earlier than floor-reared quails. These findings were supported by Batkowska et al. (2014), who reported that deep litter-reared birds performed worse than cage-reared birds in terms of early egg laying, early attainment of the age at 50 % egg production, and higher (*P <* 0.05) egg production, and Narinc et al. (2013), who also supported that the mean age at first egg was 38.9 days, and the age at 50 % egg production was 45.3 days. Apart from housing, diet also had a significant influence on sexual maturity which has been shown in the current study, birds reared with CLD attained sexual maturity faster and reached 50 % egg production earlier than those fed ED. This finding is consistent with Batkowska et al. (2014), according to their report the energy-restricted feeding delayed the development of sexual organs at ages 18, 20, and 22 weeks.

According to the present study, rearing system and diet had impact on the laying performance of Japanese quail, particularly in terms of their HDEP and HHEP. The study found that the laying performance of quails was higher in cage systems than floor, similarly, the trial with CLD showed higher performance than ED. These findings are consistent with the studies by Karousa et al. (2015), who reported that the overall egg production percentage in battery cages was significantly greater (63.54±1.68 %) than in floor systems (46.67±1.68 %) (*P <* 0.001), and also by Roshdy et al. (2010) who found that egg production for quails raised in battery cages was higher than those raised on littered floors. Other findings such as Dong et al. (2017) observed that hens in cage-rearing system had higher hen-day egg output (*P* = 0.00) than the net-rearing system and free-range system, but they were comparable. Additionally, our research remained consistent with previous research by Voslarova et al. (2006) who used ISA Brown hybrid and found increase the number of eggs, the number of eggs per hen per day. Furthermore, Ancona laying hens—a pure breed in Italy—are superior to those raised in an organic manner when it comes to laying performance when they are housed in cages (Mugnai et al., 2009). Conversely, some studies found that the rearing strategy had no effect on the number of eggs produced by commercial Shaver White hens (Neijat et al., 2011) or Lohmann Brown hens (Ahammed et al., 2014). As mentioned by Rakonjac et al. (2014), diverse hen genotypes and environmental factors (such as temperature and humidity) may contribute to varying responses in laying performance as a result of rearing systems. Moreover, Batkowska et al. (2014), observed that Japanese quails between the ages of 11 and 14 weeks had the highest HDEP and HHEP, which were significantly (*P <* 0.01) influenced by dietary protein combinations. Similarly, Yerturk et al. (2007) reported that the percent HDEP for Japanese quail fed ad libitum control, fed ad libitum daytime (07 to 17 h), and fed ad libitum night time was 80.09±2.02, 75.91±1.66, and 82.94±2.30, respectively (17 to 07 h). Additionally, Sehu et al. (2005), investigated the effects of diets with different energy and protein levels on egg production in Japanese quail from 7 to 19 weeks of age and found that the egg production percent was 84.88, 82.31, 84.12, and 85.11 for quails maintained with energy-protein levels (Kcal/kg metabolizable energy/crude protein percent) of 2657/16.68, 2654/19.75, 3010/16.45 and 2990/19.50, respectively. The results suggest that dietary protein and energy levels can impact the laying performance of Japanese quail.

Then our study has been revealed that the rearing system and diet significantly affected feed consumption (gm/bird) and feed efficiency (egg mass/kg) of Japanese quail. The birds in the cage rearing system consumed more feed and had higher feed efficiency compared to those reared on the floor. Similarly, the birds in the CLD trial consumed feed at a higher rate and had higher feed efficiency than the ED, possibly due to the palatability of the CLD diet. The higher feed efficiency observed in the CLD could be attributed to the high laying performance of the birds. Previous studies have also shown that the rearing system and diet impact feed consumption and feed efficiency in Japanese quail. Razee et al. (2016), reported that the rearing system significantly influenced Japanese quails' feed intake, with caged birds showing higher feed consumption and better feed utilization but Dong et al. (2017), showed disaccord with the current findings, according to their observations hens raised in cages had the lowest feed conversion ratio (FCR) (*P* = 0.01)

In this study, quails were housed in cages and permitted to feed ad libitum. The confined environment led to increased feed intake, contributing to enhanced feed efficiency which is not in agreement with Badawi (2017) and Sangilimadan et al. (2012) who reported that the housing arrangement had no significant impact on the Japanese quail's feed consumption rate (FCR), but get support by Yildiz et al. (2004) and Mugnai et al. (2009), who reported a feed conversion ratio (kg feed/kg egg) of 3.89 in Japanese quail that were 8 to 12 weeks old. The diet also plays a crucial role in feed efficiency, as reported by Dahouda et al. (2013), who found that the highest FCR values were recorded in the diet containing fish meal at 25 %.

The housing systems did not affect the livability percentage in this study except for some specific periods. This finding was not supported by Hanafy (2010), who reported that using floor pens reduced embryo mortality. Batkowska, Batkowska et al. (2014), reported that birds kept in cages had a higher mortality rate than those kept in pens. However, Padmakumar et al. (2000) found that the housing system had little effect on livability percentage in Japanese quails. Additionally, the diet also impacted livability, with a lower mortality rate observed in the CLD trial compared to the ED trial. Hassan et al. (2003) reported that Japanese quail after feed restriction between 2 and 5 weeks showed a mortality rate of 2.8 %, 2.8 %, and 5.6 % from 6 to 13 weeks of age for quail under 0 % and 15 % feed restrictions, and no significant difference in mortality was observed between the treatments.

The present study also observed that the external and internal quality of Japanese quail eggs was influenced by the housing and dietary systems. Internal characteristics, such as egg weight were higher in cage than floor. The literature has contradicting information about egg weight (Rakonjac et al., 2014). Studies conducted by Hidalgo et al. (2008), for example, found that eggs from conventional cage systems typically weighed less than those from free range systems. Conversely, Leyendecker et al. (2001), reported that egg weight was higher in cage systems than in floor or free-range systems. Similar results found by Minelli et al. (2007) and Krawczyk (2009) who found that the average egg weight of birds raised in either an organic or free range system was lower. Some earlier studies also have shown that heavier eggs were found in litter systems than in cages (Pištěková et al., 2005; Zemková et al., 2007). Vlčková et al. (2019), reported that the cage system had a higher internal egg index than the litter system. While it was comparable in the net-rearing and free-range systems, the egg mass (*P* = 0.00) was higher in the cage-rearing system. The free-range raising technique produced the lowest egg weight (*P* = 0.02), yolk weight (*P* = 0.00) and yolk ratio (*P* = 0.01), as well as the lowest feed intake (*P* = 0.01) (Dong et al., 2017). Yilmaz Dikmen et al. (2017), investigated the floor system and found that it had a higher yolk weight, albumen weight, albumen index, and Haugh unit, but was comparable to the conventional and enhanced cage systems. Samiullah et al. (2014), reported that the Haugh unit of eggs was higher in a conventional cage system than in a free-range system, but Đukić Stojčić et al. (2012) found that the Haugh unit in Japanese quails did not change between housing schemes. Baumgartner et al., 2008a, discovered no significant differences in egg quality between battery cages and deep-litter systems, although there were no significant differences in other internal egg quality parameters.

Regarding the external egg index the current study observed that, egg length from the cage system was significantly higher than that from other systems and get agreement with the findings of Şekeroğlu et al. (2010), who found that eggs from cage systems had a higher egg external index compared to eggs from litter systems. Elsayed and Gharib (2019) found that egg surface area and width were higher in cages than in other systems, although the differences were not statistically significant. In another study, Yilmaz Dikmen et al. (2017), also reported that the floor system with fine sawdust had the best index with significant variations. In contrast, some studies, such as Ahammed et al. (2014), and Đukić Stojčić et al. (2012), reported no impact of housing system on egg shape index, which is consistent with the present findings. Şekeroğlu et al. (2010); Van Den Brand et al. (2004); Yilmaz Dikmen et al. (2017) reported that the housing system did not affect egg shell quality, which is inconsistent with the present findings. El-Sheikh et al. (2016), found that the housing system (cage & floor) had no significant effect on the percentage of shells and the thickness of those shells with or without membranes.

In a study conducted by Franco et al. (2020), it was observed that laying hens fed with commercial feed exhibited higher weight and thicker eggshells compared to those fed with corn/pea/triticale and corn/wheat-based diets. However, there were no significant differences in albumen height, eggshell weight, and haugh units among the different diet types. On the other hand, Kubiś et al. (2018) reported comparable differences in egg weight and shell thickness between a diet based on white lupine and a control diet based on corn and soy. Ekeocha et al. (2021), observed significant differences (*P <* 0.05) in egg height, egg breadth, yolk weight, yolk height, albumen weight, albumen height, Haugh unit, and eggshell index among hens fed on handmade feed and those fed on commercial feed.

The housing system did not significantly impact the chemical composition of egg albumen and egg yolk. This is consistent with the findings of Matt et al. (2009), who reported no discernible differences in the fatty acid, protein, or dry matter content of eggs between cage and floor-rearing systems. Dong et al. (2017) also mentioned in their observation that very little impact of housing system on the quality of hen eggs. In case of dietary effect, all the analyzed parameters had significantly higher values in the commercial diet than the experimental diet. Islam et al. (2021) also demonstrated that changes in diet substantially impacted the higher egg quality such as dry matter, crude protein, crude fiber, and ash percentages in ready-made diet.

## 5 Conclusion

The results of this study show that commercial feed and living circumstances have a major impact on the quantity, composition, and quality of Japanese quail eggs. Birds kept in cages demonstrated early maturation as well as higher efficiency, feed consumption, and egg production. Livability and a few chemical characteristics, however, did not change. Similar gains were seen in birds fed a commercial diet, including improved egg content. The results, in spite of the difficulty of managing vices and availability of commercial feed, justify the rearing of Japanese quail in cages using commercial diets. In order to determine whether the commercial diet is feasible, more research on the fatty acid profile of eggs from quails fed commercially is required.

## Ethical approval

The authors of this study only used quail eggs and followed the approved practices for raising birds in Bangladesh, which include cage and floor rearing. This study did not require ethical approval due to the following conditions and in compliance with the policy of the Department of Poultry Science at Sylhet Agricultural University.

## CRediT authorship contribution statement

**Md. Amir Hossain:** Writing – original draft, Software, Resources, Methodology, Investigation, Funding acquisition, Formal analysis, Data curation, Conceptualization. **A.S.M. Mahbub:** Writing – review & editing, Supervision, Project administration, Methodology, Data curation, Conceptualization. **Shah Ahmed Belal:** Writing – review & editing, Software, Methodology, Investigation, Formal analysis, Conceptualization.

## Declaration of competing interest

The authors have no conflicts of interest to declare. All co-authors have seen and agree with the manuscript's contents, and there is no financial interest to report. We certify that the submission is original work and is not under review at any other publication.
